# High-Volume Hemodiafiltration Versus High-Flux Hemodialysis: A Narrative Review for the Clinician

**DOI:** 10.3390/jcm14082614

**Published:** 2025-04-10

**Authors:** Bernard Canaud, Giovanni Strippoli, Andrew Davenport

**Affiliations:** 1 School of Medicine, Montpellier University, 9 Rue des Carmelites, 34090 Montpellier, France; 2 Department of Precision and Regenerative Medicine and Ionian Area (DIMEPRE-J), The University of Bari, Piazza Umberto I, 70121 Bari, Italy; 3 UCL Department of Renal Medicine, Royal Free Hospital, University College London, London WC1E 6BT, UK

**Keywords:** end-stage kidney disease, kidney replacement therapy, dialysis, patient outcomes, high-volume hemodiafiltration, convective dose

## Abstract

**Background:** End-stage kidney disease (ESKD) management presents a significant challenge, with increasing patient burden, escalating costs, and unmet needs in improving survival and quality of life. High-volume hemodiafiltration has been found to offer enhanced solute clearance, improved inflammatory marker profiles, and better patient-centered outcomes in multiple trials compared with high-flux hemodialysis. Recent data also confirm a survival advantage compared to standard high-flux hemodialysis. **Methods:** We compiled a narrative review for the clinician illustrating evidence supporting the comparative performance of high-volume hemodiafiltration with conventional high-flux hemodialysis in ESKD management. Data on intermediary outcomes including biochemical and clinical benefits, as well as patient-centered outcomes and all-cause and cardiovascular death data from prospective randomized trials, their meta-analyses, and real-world cohort studies were reviewed and summarized. **Results:** Randomized studies in adults have found that high-volume hemodiafiltration demonstrates superior outcomes, with a 23% improvement in survival rates when achieving convective volumes ≥23 L/session, enhanced removal of uremic toxins, reduced inflammation, and better patient-reported outcomes. Cohort studies in pediatric populations find associations with improvements in growth, cognitive development, and cardiovascular health. Hemodiafiltration appears to be cost-effective when accounting for extended life expectancy and improved quality of life, although the existing data are limited to European geographies. **Conclusions:** Hemodiafiltration offers enhanced survival, a reduced treatment burden, and improved quality of life for ESKD patients. Given the existing data of superiority versus high-flux hemodialysis, it is plausible that hemodiafiltration will become the standard of care.

## 1. Introduction: Hemodiafiltration as a Kidney Replacement Therapy Option

The management of end-stage kidney disease (ESKD) presents a significant burden on healthcare systems due to the continually rising number of patients, despite the implementation of various kidney protective measures [1]. This burden is further exacerbated by the complexity of patient care, associated costs, and the ongoing need to improve patient outcomes, including patients’ quality of life and perception of care.

The effective management of ESKD requires multiple strategies, relying on three key pillars of treatment, including kidney disease and symptomatic management, dialysis, and kidney transplantation [2,3]. Optimizing individual patient management depends on patient profiles, disease trajectories, available resources, and the structure of the healthcare system [4]. Kidney protection, conservative management, dialysis, and transplantation are all essential and complementary components of this comprehensive approach [5].

Within this context, dialysis plays a crucial role as a pillar of kidney replacement therapy, including various modalities; hemodialysis, hemodiafiltration, and peritoneal dialysis [6,7]. Recent high-quality evidence, including prospective randomized trials and meta-analyses, has identified the superiority of high-volume hemodiafiltration over conventional high-flux hemodialysis [8,9,10].

The aim of this narrative review is to summarize evidence on the benefits and harms of high-volume hemodiafiltration compared with the current standard of care, which is represented by high-flux hemodialysis. We approached this in the interest of clinicians who may use this as an overview [11]. We attempted to do this using a framework for presenting data conceptualized by Sackett et al., which incorporates three key domains: scientific evidence, patient perception, and clinical judgment, allowing for a holistic assessment of treatment value, when including treatment costs.

## 2. Evidence-Based Medicine and Real-World Evidence Applied to Hemodiafiltration: A Clinician Perspective

The nephrology community widely acknowledges the importance of integrating solid scientific data with clinician experience and patient perspectives in the management of patients with ESKD [12,13,14]. Physicians must critically evaluate evidence through their own experience and must balance findings from diverse studies and clinical case scenarios in order to optimize individual patient care. This principle is crucial when considering the relative benefits of hemodiafiltration (HDF) compared to standard hemodialysis (HD). In this section, we explore the key issues affecting the evaluation of evidence comparing HDF and HD.

1. ***Time Period of Assessment Issue: ***Before 2010, randomized interventional studies on HDF and other convective therapies were limited by outdated technology, including inadequate allocation concealment, issues with blinding, failure to use intention to treat analysis, insufficient focus on HDF alone, convective dose delivery, and small sample size. These studies typically also had inadequate statistical power to assess hard clinical endpoints. Meta-analyses attempt to aggregate data from the existing trials often included trials on various forms of convective-based therapies, post-dilution and pre-dilution HDF, acetate-free biofiltration, hemofiltration, and paired hemofiltration, and were not able to standardize the convective dose delivered [15,16]. These findings have now been updated by the generation of new and more timely evidence.
2. ***Threshold Convective Dose Issue*:** The importance of the convective dose was further emphasized by an individual patient data meta-analysis conducted in 2016, which demonstrated that the total convective dose delivered plays a critical role in patient outcomes [17,18]. This study highlighted a dose-dependent survival advantage for HDF versus HD, with improvements beginning at 23 L and extending up to 30 L of substitution volume per session. Building on this knowledge, the comparison of high-dose hemodiafiltration with high-flux hemodialysis (CONVINCE) study definitively established that the clinical benefits of HDF are directly linked to the convective dose delivered during each session [19]. For the standard European patient as included in the CONVINCE trial, a minimum threshold of 23 L of substitution volume per session was used to achieve the significant clinical benefits shown. Studies should account for this threshold.
3. ***Patient-Reported Outcome (PRO) Issue: ***Previous studies evaluating patient-reported outcomes (PROs) and health-related quality of life (HRQoL) in HDF were limited by several factors including a limited number of patients recruited, low sensitivity of assessment tools (for example, short form 36 (SF-36) and EuroQol 5 Dimension (EQ-5D) questionnaires), infrequent assessment points, and failure to achieve the optimal targeted convective dose [20,21,22]. This highlighted the need for a more robust, well-designed, and adequately powered study to provide meaningful insights, which was the basis of CONVINCE.
4. ***Cost-Effectiveness and Cost-Utility Issue: ***Early cost-effectiveness studies failed to account for adequate convective dosing in HDF, akin to assessing the efficacy of a drug at subtherapeutic doses [23,24]. An evaluation of the economic value of HDF also requires studies that perform an assessment with the delivery of a specific convective dose. The CONVINCE study set a new benchmark by incorporating both cost data and quality-of-life assessments. This comprehensive approach provides a more robust evaluation of the healthcare economic value of high-volume HDF compared with conventional high-flux HD, addressing the shortcomings of earlier analyses and offering a more accurate assessment of cost-effectiveness and utility.
5. ***Real-World Evidence Issue: ***Real-world evidence is increasingly recognized as essential for evaluating new therapies, particularly regarding generalizability and applicability in broader unselected populations [25,26]. This approach is especially relevant in ESKD patients and in communication with clinicians, who often argue that randomized trials preselect patients, do not necessarily reflect the current dialysis population, and apply strict protocols and practices with additional close monitoring that may then introduce serious biases to generalizability [27,28]. This type of evidence will also be of particular relevance when the prospect of designing and conducting new trials of HDF appears to be low, but these may be still required for policy change in geographies or selected populations which have not been previously involved in randomized trials.

## 3. Transitioning from High-Flux Hemodialysis to High-Efficient or High-Volume Hemodiafiltration

HDF was originally introduced to expand the removal of uremic toxins beyond small molecular weight (MW) compounds and enhance hemodynamic stability during dialysis [29]. To achieve these goals, the concept of the online production of sterile, non-pyrogenic substitution fluid emerged, made possible through a cold sterilization process using sterilizing ultrafilters. This advance was further supported by the development of dedicated HDF machines equipped with advanced features, including precise fluid balance; automated ultrafiltration control; dialysate flow-blood flow alignment; automated priming, rinsing, and bolus administration during treatment as needed [30]. However, the importance of achieving a higher convective dose with HDF has only been recently established, with studies, including the landmark CONVINCE trial, demonstrating that a minimum substitution volume of 23 L per session (approximately 13 L/m^2^ body surface area), or 66 L per week in a thrice-weekly schedule, significantly reduced mortality risk [18,19]. Optimizing the prescription of hemodiafiltration (HDF) is increasingly recognized as essential to delivering the highest treatment efficiency while minimizing the environmental impact, particularly in terms of dialysate and water consumption. High-volume HDF, when appropriately prescribed, is emerging as the most sustainable and eco-friendly kidney replacement therapy. Achieving this requires a careful and systematic approach. Blood flow should be increased to at least 350 mL/min whenever possible, and the high-flux capillary dialyzer should be selected to maintain an optimal surface area–blood flow ratio, typically around 1.0 m^2^ per 200 mL/min. The total dialysate flow should align with the blood flow, aiming for a dialysate–blood flow ratio of approximately 1.2. Ultrafiltration flow rate should be optimized to achieve a filtration fraction between 30% and 35%, either through manual adjustments or, preferably, by utilizing automated functions embedded in HDF machines. For example, in an 80 kg patient receiving a 4 h treatment with a blood flow of 350 to 400 mL/min, an appropriate high-flux dialyzer would have a surface area between 1.8 and 2.0 m^2^, the total dialysate flow would be set at 400 mL/min, and the substitution flow rate would range from 100 to 110 mL/min (filtration fraction of 30 to 35%), corresponding to a substitution volume of 24 to 26 L per session. Net ultrafiltration for weight loss would typically be between 2 and 2.5 L. Under these conditions, a spKt/V of around 1.7 and a β2-microglobulin reduction ratio of approximately 85% can be expected. Anticoagulation should be adapted to individual patient needs using either standard unfractionated heparin or low-molecular-weight heparin, preferably administered on the venous line for optimal efficacy. Modeling studies, supported by real-world data from a large dialysis provider, suggest that such a prescription strategy offers an optimal balance of HDF efficiency, safety, and sustainability [31,32].

## 4. Clinical Outcomes: Intermediary and Explanatory Outcomes

Hemodiafiltration has been shown to be associated with a series of beneficial effects on biochemical and clinical endpoints compared with high-flux HD. They are briefly summarized in this section.

### 4.1. Biochemical Effects of Hemodiafiltration (HDF)

**Uremic Solute Removal: Small Molecules**: HDF increases the clearance of small molecules including urea and phosphate by 10–15% compared to conventional HD [33,34,35]. This enhanced removal contributes to lower pre-dialysis serum phosphate levels and reduced phosphate binders’ requirements, supporting better phosphate management [36]. **Middle Molecules:** HDF provides superior clearance of middle-molecular-weight toxins, for example, β2-microglobulin (MW 11,800), resulting in a 10–20% reduction in pre-dialysis serum levels and a more substantial reduction in time-averaged concentrations [37,38]. This improvement has been associated with reduced mortality, particularly cardiovascular-related deaths and infections [39,40,41,42,43]. **Large Molecules**: HDF enhances the removal of various large molecules (MW 15–45 kDa) encompassing complement factor D, leptin, fibroblast growth factor-23 (FGF23), and various cytokines [44,45,46,47,48,49]. These toxins are implicated in inflammatory responses, nutritional imbalances, and metabolic bone disorders, contributing to overall health improvement.

**Protein-Bound Uremic Toxins**: However, the protein-bound toxins indoxyl sulfate and *p*-cresyl sulfate are not particularly well removed by HDF as their free fraction is limited (10 to 15%), with some studies showing a modest reduction (5–10%) in serum levels [50,51].

**Inflammation and oxidative stress:** There is a broad consensus that HDF reduces inflammation and oxidative stress markers in patients with ESKD [52,53,54]. Recent pediatric studies, including the HDF heart and height study, have shown that HDF leads to significant reductions in key inflammatory markers including C-reactive protein (CRP) and interleukin-6 (IL-6), as well as the oxidative stress indicators thiobarbituric acid reactive substances (TBARSs) and advanced oxidation protein products (AOPPs) [55,56]. Remarkably, these improvements were evident after just three months of treatment and then sustained over extended follow-up periods. The reduction in inflammation and oxidative stress has been associated with improved nutritional status, enhanced growth and developmental outcomes, and better cardiovascular health in children.

### 4.2. Clinical Effects of Hemodiafiltration (HDF)

*Dialytic Symptomatology and Dialysis Tolerance*: HDF significantly reduces intradialytic hypotensive (IDH) episodes and reduces the more common dialysis-related symptoms of cramps, headaches, and vomiting. These benefits have been variously attributed to the negative thermal balance from cooler infusion fluids, improved removal of vasodilatory mediators, and enhanced endothelial function [57,58,59,60]. However, when thermal and fluid management are comparable, then the hemodynamic advantage of HDF may be less apparent [61]. Nevertheless, improved hemodynamic stability may contribute to better cardiovascular health [62,63].

*Patient Perception and Patient-Reported Outcomes*: The results from earlier randomized trials of HDF on HR-QOL were mixed. Some reported significant improvements in physical and social functioning [64], while others found no notable changes [65,66]. Meta-analyses suggested better social functioning and reduced fatigue [64]. The CONVINCE study, which employed the PROMIS tool, which assessed physical, mental, and social domains every 3 months over an extended period, provided robust data to show that treatment with HDF led to better cognitive and physical function and social interaction than standard high-flux HD [67].

*Anemia Management and Erythropoietin-Stimulating Agent (ESA) Use*: Prospective trials and cohort studies, including the RISchio CArdiovascolare nei pazienti afferenti all’ Area Vasta In Dialisi (RISCAVID) and the role of hemodiafiltration on ERI (REDERT), have demonstrated that HDF reduces the erythropoietin resistance index (ERI), improving anemia management [68,69,70]. This effect may be due to the removal of erythropoietic inhibitors, decreased inflammation, and better iron utilization driven by reduced hepcidin levels [71,72,73].

*Nutritional Status and Body Composition*: HDF has been associated with improved appetite, better dietary protein intake, and the preservation of lean body mass [74,75]. The visceral protein markers albumin and prealbumin generally remain stable [76]. Although HDF may cause slight increases in the loss of amino acids, water-soluble vitamins, and trace elements, appropriate oral supplementation can help maintain nutritional balance [77,78].

*β2-Microglobulin Amyloidosis*: Historical cohort studies suggest that HDF reduced the incidence of β2-microglobulin amyloidosis, including carpal tunnel syndrome and related complications [79]. This improvement probably results from a combination of enhanced β2-microglobulin clearance, the use of ultrapure water, and more biocompatible dialysis materials [80].

*Bone Mineral Disorders*: HDF improves phosphate removal, lowering parathyroid hormone (PTH) and fibroblast growth factor (FGF23) levels. Combined with a positive calcium balance, this effect promotes healthier bone turnover in children and by increasing nanocalciparticle clearance may potentially reduce vascular calcification [81,82].

*Residual Kidney Function Preservation*: Preliminary cohort studies found that HDF might preserve residual kidney function over and above conventional HD [83,84]. Although data are still suboptimal, this matter deserves further exploration as it could facilitate the adoption of incremental kidney replacement therapy, offering a gentler transition into dialysis treatment. Further research is warranted in this aspect of clinical care which is of high relevance to the patients.

## 5. Clinical Outcomes: Hard Clinical Endpoints

To ensure consistency and interpretability in studies investigating HDF, three key factors must be considered: the time period of the study, the specific HDF treatment modality, and the sessional convective volume. Prior to 2006, clinical evidence supporting the benefits of HDF was limited. The turning point came with the Dialysis Outcomes and Practice Patterns (DOPPS) study in 2006, which reported that HDF was associated with reduced all-cause mortality [85,86], although in cohort models. Between 2006 and 2014, four European randomized controlled trials (RCTs) were conducted [10,57,58,87,88,89]. These trials yielded inconsistent results, with only the Estudio de Supervivencia de Hemodiafiltración On-Line (ESHOL) study delivering high convective volumes, demonstrating clear superiority [90]. During the same period, the findings from cohort studies and national registries suggested the clinical benefits of HDF compared to HD. In 2016, an individual patient data (IPD) meta-analysis from the European HDF Pooling Project identified convective dose as a key factor driving improved outcomes [17,18]. The 2023 CONVINCE study provided further clarity, experimenting and confirming the hypothesis that HDF was superior to high-flux HD when convective volumes exceeded 23 L per session [19,22]. In 2024, secondary outcomes of the CONVINCE study, particularly patient-reported outcomes, confirmed that HDF improves several aspects of patient-relevant health status [67]. Additionally, an updated IPD meta-analysis including over 4000 patients reinforced the survival advantage conferred by HDF over the current standard of care which is high-flux HD [8]. In short, HDF is superior to conventional high-flux HD for survival and cardiovascular endpoints. This has been demonstrated by both individual trials (CONVINCE) and their pooled analyses (European HDF Pooling Project).

### 5.1. Randomized Controlled Trials

Following the findings of the DOPPS study in 2006, the hypothesis generated of an association of exposure to HDF with improved survival was tested in six key European RCTs evaluating HDF. These were designed, conducted, and reported between 2012 and 2023, and summarized in Table 1.

The Italian Convective study was a multicenter, randomized controlled trial that recruited 146 HD patients (70 HD, 36 HF, 40 HDF) [57] to assess the impact of convective therapies (HF and HDF) on intradialytic symptomatic hypotension (ISH) and vascular stability compared to HD. HDF reduced ISH by 50.9% (*p* < 0.001) and HF by 18.4% (*p* = 0.011), while HD saw a slight increase. Pre-dialysis systolic BP increased significantly in HDF patients. The CONvective TRAnsport STudy (CONTRAST) randomized 714 to -HDF or low-flux HD with an average convective volume of 20.7 L/session [87,88]. While no significant differences were observed in all-cause mortality or cardiovascular events, the on-treatment analysis revealed a survival benefit for patients receiving high-volume HDF (>21.95 L/session). The Turkish online HDF Study randomized 782 patients to -HDF or high-flux HD, with a mean convective volume of 17.2 L/session [88]. Although no significant differences were found in primary outcomes, high-efficiency -HDF (>17.4 L/session) reduced all-cause mortality by 46% and cardiovascular mortality by 71%. The ESHOL study enrolled 906 patients, and those treated with HDF (22.9–23.9 L/session) experienced a 30% reduction in all-cause mortality and a 33% reduction in cardiovascular mortality [90]. Additionally, infection-related mortality and intradialytic hypotension episodes were lower with HDF. The French Convective versus Hemodialysis in Elderly (FRENCHIE) Study enrolled 381 elderly patients (>65 years), with a mean convective volume of 20.0 L/session [58]. Although mortality and health-related quality of life outcomes were similar between groups, HDF improved intradialytic tolerance, β2-microglobulin clearance, and metabolic bone markers.

Despite these RCTs, the results were inconsistent, with only the ESHOL study, delivering high convective volumes, demonstrating clear superiority. The reason had to do primarily with inadequate sample sizes and highlighted the need to investigate the role of practice patterns and particularly the convective dose in determining clinical benefits both in pooled analyses and in properly sampled randomized trials. This led to the European HDF Pooling Initiative, which conducted an individual patient data (IPD) meta-analysis [17,18]. This finding served as the foundation for designing the CONVINCE trial, a landmark study aimed at conclusively evaluating the benefits of high-dose HDF for survival, which randomized patients to high-volume HDF versus high-flux HD.

The CONVINCE trial was a pragmatic, multinational, European RCT investigating the effects of high-dose HDF compared to conventional high-flux HD in ESKD patients [22] funded by the European Union as part of the Horizon 2020 innovation program (grant 754803). A total of 1360 patients were enrolled, with 683 high-flux HD patients assigned to HDF and 677 to remain on high-flux HD. The median follow-up period was 30 months, and patients in the HDF group received a mean convective volume of 25.3 L per session. The primary outcome was all-cause mortality, while the secondary outcomes included cause-specific mortality, composite cardiovascular outcomes, kidney transplantation rates, and recurrent hospitalizations. High-dose HDF (with substitution volumes exceeding 23 L/session) significantly reduced all-cause mortality compared to HD, with a hazard ratio (HR) of 0.77 (95% CI: 0.65–0.93; *p* = 0.005). Infection-related mortality, including deaths related to COVID-19, was also lower in the HDF group (HR: 0.69; 95% CI: 0.49–0.96). However, no significant differences were observed in cardiovascular mortality or recurrent hospitalization rates. It is important to note that the study was conducted during the COVID-19 pandemic, which potentially contributed to some misclassification of the causes of death, particularly among patients hospitalized with COVID-19. Despite this limitation, the key finding was that high-dose HDF provided a survival advantage compared to high-flux HD, particularly when substitution volumes exceeded 23 L per session. These results emphasize the importance of achieving sufficient convective doses to optimize clinical outcomes.

The High-Volume Hemodiafiltration vs. High-Flux Hemodialysis Registry Trial (H4RT) is another pivotal ongoing RCT exploring the clinical benefits of HDF over HD [91]. This UK-based registry trial, which is still ongoing, aims to provide further insights into the comparative effectiveness of high-volume HDF, defined as targeting a substitution volume of more than 23 L per session in post-dilution mode, compared to high-flux HD with results anticipated by 2026.

### 5.2. Meta-Analyses and Individual Patient Data Meta-Analyses

Early meta-analyses conducted prior to 2016 focused on various convective dialysis modalities other than HDF, including HF, acetate-free biofiltration (AFB), and paired filtration dialysis (PFD) [92,93,94,95,96,97,98,99]. However, these studies did not specifically address HDF only, as they were large-scale systematic reviews looking at convection versus diffusion as a whole. There also was no possibility to consider the actual convective dose delivered, which the CONVINCE study found to be an important element in the survival advantage as the experimental intervention in CONVINCE was high-volume HDF. In contrast, another meta-analysis by Mostovaya which only included HDF studies demonstrated significant survival benefits, highlighting the need for a more targeted approach when analyzing this modality, although having a number of methodological limitations [99]. To further illustrate these findings, a summary table of the meta-analyses is presented in Table 2.

More compelling evidence comes from a 2016 individual patient data meta-analysis (IPD-MA), which addressed the limitations of earlier studies, as shown in Table 3. These findings that have been confirmed by the most recent 2024 IPD-MA, which includes data from over 4000 patients and is presented in Table 4.

These analyses emerged from the HDF European Pooling Project, with the first individual patient data meta-analysis published in 2016 [17,18] including over 2793 patients, and an updated 2024 version incorporating data from more than 4096 patients [8]. Both studies confirmed the clinical benefits of HDF, reporting a 20% reduction in all-cause mortality and a 14% reduction in cardiovascular mortality.

Additionally, these analyses revealed that convective dose played a critical role in improving outcomes, with the benefits observed in a dose-dependent manner [8]. Interestingly, no significant reductions were noted in infection-related mortality or hospitalization rates. Although subgroup analyses were somewhat limited, certain populations, such as the elderly, cardiac, and diabetic patients, appeared to derive enhanced benefits from HDF.

### 5.3. Cohort Studies and Real-World Data Studies

Cohort studies provide real-world insights into the effectiveness of online HDF [83,85,100,101,102,103,104,105]. The summarized data on adult ESKD patients in Table 5 (A: cohorts; B:national registry cohorts) illustrate a clear trend demonstrating an advantage for HDF. Before 2006, only three studies explored survival with HDF versus HD, with neither demonstrating significant positive associations [79,100,101]. The landmark 2006 DOPPS study was the first large, prospective study to report a 23% lower all-cause mortality with high-efficiency HDF [84]. This finding was corroborated by a Fresenius European database (EuClid) analysis [85]. However, a recent analysis of newer DOPPS phases failed to confirm these benefits [106], which is inherent when using observational study designs to address intervention questions. These studies can only show associations, which may be confounded by multiple factors, such as inconsistent identification of convective modalities and inadequate reporting of convective volumes. 

Numerous national and large cohorts in France, Australia/New Zealand, and Japan have consistently reported better associations with survival for ESKD patients who were treated with HDF than standard HD. The French National Registry (REIN) observed that HDF was associated with higher survival, with a hazard ratio (HR) for all-cause mortality of 0.84 and cardiovascular mortality of 0.73 [107]. Facilities with higher HDF usage reported even lower mortality rates. The Australia and New Zealand Dialysis and Transplant Registry (ANZDATA) published reduced all-cause mortality for HDF-treated patients, with HRs of 0.79 in Australia and 0.88 in New Zealand, along with lower cardiovascular mortality in the Australian cohort (HR 0.78) [108]. The Japanese Society for Dialysis Therapy (JSDT) registry noted that predilution HDF was associated with reduced all-cause mortality (HR 0.83) and lower cardiovascular mortality when high-volume HDF was used [109]. Additionally, recent propensity score-matched cohort studies from Latin America (Brazil and Colombia) demonstrated that HDF was associated with a reduction in all-cause mortality, with HRs of 0.71 and 0.45, respectively [103,104]. Furthermore, a large international cohort study of 85,222 dialysis patients from a major kidney care provider, with more than 50% of the patients receiving HDF, reported an all-cause mortality HR of 0.78 [105].

Positive associations between HDF and improved outcomes have also been repored in pediatric ESKD patients [48,55,110]. Table 6 summarizes findings from several pediatric ESKD cohorts comparing the outcomes of HDF versus conventional HD.

In summary, real-world evidence from multiple countries shows that HDF is associated with an average lower risk of all-cause mortality by 18% (interquartile range: 16–21%). This coherence of many cohort studies is a hint to a potential benefit that can and should be, and has been, demonstrated in prospective randomized trials.

**Table 5:** **A.** Real-world evidence: cohort studies comparing the outcomes of HDF and HD in adult ESKD patients from 1992 to 2024. **B.** Real-world evidence: national cohort studies comparing the outcomes of HDF and HD in ESKD adult patients.

**A.**						
**Author, Journal Year**	**Study Design**	**Population**	**n Patients**	**Key Features**	**Substitution Volume (HDF)**	**Main Outcomes**
Kerr et al., (KI, 1992) [100]	Retrospective Study (France)	20 Prevalent patients	HD 20, HDF 20	18-month follow up	15–25 L/session (estimated)	Improved clearance of small and middle molecules with HDF;
						No significant change on mortality
Locatelli et al., (KI, 1999) [79]	Observational Cohort Lombardy Registry	7380 Prevalent patients	HD 6298, HDF 1082	29.7-month follow-up	Not specified	10% non-significant mortality reduction with HDF/HF
Canaud et al., (KI, 2006) [85]	Prospective Cohort (Euro-DOPPS)	2165 Prevalent patients	HD 1912 HDF 253	3-year follow-up	15–25 L/session (estimated)	35% mortality reduction with high-efficiency HDF (adjusted)
Vilar et al., (CJASN, 2009) [83]	Retrospective Cohort (UK)	858 Incident patients	HD 626 HDF 232	over 18 years	15–25 L/session (estimated)	34% mortality reduction with HDF/HD
Canaud et al., (KI, 2015) [102]	Retrospective Cohort (EuClid, Europe)	2293 Incident patients	HDF 2203	Examined optimal convection volumes for OL-HDF	18–25 L/session	Reduced mortality as function of convective volume;
						Reduction in ß2M and CRP levelsas function of convective volume;
Valderrama et al., (KD, 2022) [103]	Retrospective Cohort + PSM (Colombia)	2361 prevelent patients from Colombia	HD 505 HDF 505	2-year follow-up	23 L/session	45% mortality reduction with HDF/HD; (14.3% mortality in HF-HD vs 5.9% in HV-HDF)
						Reduction of CV mortality in patients < 60 years.
Da Rocha et al., (JCM, 2024) [104]	Retrospective Cohort + PSM (Brazil)	149,372 prevalent patients	HD 170 HDF 85	12-month Follow-Up	23 L/session	29% mortality reduction with HDF/HD; (92.1% survival in HDF vs 79.9% in HD (1 year))
Zhang et al., ASN 2023 [105]	Real-world observational study	78,608	HD 36012 HDF42596	HDF vs HD effectiveness assessment during the COVID-19 pandemic	≥23 L/session	22% mortality reduction (all-cause) vs HD particularly during COVIC pandemic
		prevalent patients EMEA 23 countries (EuClid)				17% hospitalization admission with HDF/HD
**B.**						
**Author, Journal Year**	**Design**	**Population**	**Number of Patients**	**Key Features**	**Substitution Volume (HDF)**	**Main Outcomes**
Kikuchi et al. (KI 2018) [109]	Observational, propensity-matched cohort study	Predilution HDF and HD patients in Japan	10,784 HDF, 227,972 HD	Predilution HDF with high and low substitution volumes; 1-year follow-up	Low Substitution Volume 25.1 ± 9.4 L/session High Substitution Volume 50.3 ± 10.2 L/session	Predilution HDF associated with lower all-cause mortality (HR 0.83) and cardiovascular mortality with high-volume HDF. Optimal substitution volume: 50.5 L/session.
Mercadal et al. (AJKD 2015) [107]	Observational study using the French REIN Registry	Incident dialysis patients in France	5526 HDF, 22,881 HD	Analyzed HDF use at patient and facility level; median HDF use 1.2 years	Not Reported	HDF associated with better survival: all-cause mortality (HR 0.84), cardiovascular mortality (HR 0.73).
						Facility-level HDF use improves outcomes.
See et al. (NDT 2018) [108]	Cohort study using the ANZDATA Registry (2000–2014)	Dialysis patients in Australia and New Zealand	4110 HDF, 22,851 HD	Binational (Australia/New Zealand); multivariable Cox regression; median follow-up 5.3 years	Not Reported	HDF associated with lower all-cause mortality (HR 0.79 Australia; HR 0.88 New Zealand).
						Reduced cardiovascular mortality in Australian cohort (HR 0.78).

**Table 6:** Real-world evidence: cohort studies comparing the outcomes of HDF and HD in ESKD pediatric patients.

Author, Journal Year	Design	Population	n of HD Patients	n of HDF Patients	Aim and Key Features	Primary Outcome	Secondary Outcome	Main Outcomes Results
Agbas et al.; (PLOSO 2018) [48]	Prospective observational study	ESKD Children on HD and HDF	22	22	Compare inflammation and oxidative stress in HD vs HDF	Inflammation, oxidative stress biomarkers	Endothelial function, antioxidant capacity	Reduced inflammation and oxidative stress in HDF group
Fischer et al., (KIR 2021) [110]	Multicenter longitudinal study	ESKD Children on HD and HDF	61	42	Compare bone metabolism and inflammation markers in HD vs HDF	Bone-specific alkaline phosphatase levels and inflammatory markers	Fibroblast growth factor-23/klotho ratio, bone disease progression	Higher bone formation markers, lower inflammation in HDF group
Shroff et al., (JASN 2018) [56]	Multicenter observational cohort study	ESKD Children on HD and HDF	78	55	Assess cardiovascular markers and height improvement in HD vs HDF	Carotid intima-media thickness (cIMT) and height SD score	Blood pressure, intradialytic symptoms, patient-reported outcomes	Reduced cIMT progression, increased height, better BP control in HDF

## 6. Patient Reported Outcomes

The CONVINCE study addressed many of the limitations of previous research by utilizing the PROMIS tool, a questionnaire designed to assess physical, mental, and social domains over time, and provided more robust data [22]. It also incorporated regular evaluations over a 3-year follow-up period [67]. However, while both HD and HDF were associated with declines in patient-reported outcomes (PROMs) over time, HDF showed better preservation of key domains including cognitive function, and social interaction [90]. These findings suggest that HDF enhances patient perception of treatment, promoting improved quality of life.

## 7. Cost-Effectiveness and Cost-Utility

The Canadian arm of the CONTRAST reported that HDF was a more costly treatment than low-flux HD, but the overall treatment costs were similar due to the reduction in medication costs for those treated with HDF [111]. The most recent analysis, conducted by healthcare economics experts and incorporating new data from the CONVINCE study, demonstrated that high-dose HDF increased quality-adjusted life years (QALYs) but also raised annual costs compared to high-flux HD [112]. In the two-year trial-based analysis, HDF provided higher quality-adjusted life years (QALYs) than standard HD, but at a higher cost. The incremental cost-effectiveness ratio (ICER) ranged between EUR 31,898 and EUR 37,344, depending on dialysis staff expenses. A lifetime Markov cohort model estimated ICERs between EUR 27,068 and EUR 36,751. Compared to HD, HDF offered an additional year of perfect health but with increased costs. Sensitivity analyses showed a cost-effective probability exceeding 90% at a willingness-to-pay threshold of EUR 50,000 per QALY. When excluding all costs related to additional life years, the ICER dropped to EUR 13,231. The main driver of the ICER was the cost of extra dialysis sessions during extended life years, rather than the cost per individual session [110].

## 8. Discussion and Conclusions

**Figure 1:**
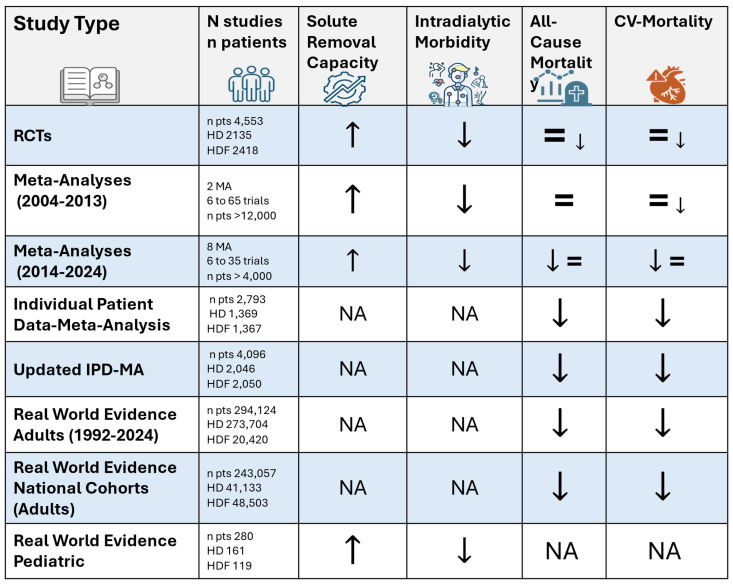
Graphical summary of major studies and key outcomes.

From a clinician’s perspective, this narrative review underscores the superior benefits of high-volume HDF for patients with ESKD. High-volume HDF surpasses high-flux HD in key intermediary outcomes, including the enhanced solute clearance of middle and large MW solutes, the improved control of uremic toxins, and the reduction in inflammation and oxidative stress. More importantly, it translates these improvements into significant clinical and patient-centered benefits. These include a 22–23% improvement in survival rates when a minimum convective dose of 23 L/session is achieved, a slower decline in patient-reported outcomes such as cognitive function and social engagement, and a cost-effective alternative modality to high-flux HD. A graphical summary of the major studies and key outcomes is presented in Figure 1. Such evidence may support a progressive paradigm shift in kidney replacement therapy, making high-volume HDF the preferred option and up to the standard of care for the majority of patients. Additionally, these benefits extend beyond adult patients, showing promising results in pediatric ESKD populations as well. However, while high-volume HDF marks substantial progress, it should not be viewed as a standalone solution. Further initiatives should focus on leveraging HDF as a customizable treatment platform, enabling personalized care that aligns with individual patient needs and regional healthcare policies.

## Figures and Tables

**Table 1:** Randomized controlled trials comparing the outcomes of HDF and HD in ESKD adult patients (2010–2023).

Author, Journal Year	Design	Population	n of Patients	Mean Sub/Conv Vol. (L/session)	Aim and Key Features	Primary Outcome	Secondary Outcome	Main Outcomes Results
Locatelli et al., (JASN 2010) [57]	Italian Multicenter RCT	Prevalent End Stage Kidney Disease Patients	146 (70 HD, 36 HF, 40 HDF)	Sub. 30-40 predilution mode	Comparison of predilution HDF and HF vs. HD on intradialytic hypotension and vascular stability	Frequency of symptomatic intradialytic hypotension (IDH)	Changes in pre-dialysis systolic BP, dropout rates, treatment survival, and adverse effects	IDH decreased by 50.9% with HDF, 18.4% with HF, and slightly increased with HD
Grooteman et al., J(ASN 2012) [86]	Dutch, Canadian Multicenterl RCT	Prevalent End Stage Kidney Disease Patients	714 (HD 356, HDF 358)	Sub. 19.8 [15,16,17,18,19,20,21,22,23,24,25,26,27,28]	Comparison pf online postdilution HDF vs. low-flux HD	All-cause mortality	Cardiovascular events, renal transplants	No significant difference in all-cause mortality (HR 0.95, 95% CI 0.75–1.20)
Maduell et al., (JASN 2013) [89]	Spanish Multicenter RCT	Prevalent End Stage Kidney Disease Patients	906 (450 HD, 456 HDF)	Sub. 21.8 Conv. 23.9	Comparison of high-volume postdilution OL-HDF vs. high-flux hemodialysis	All-cause mortality	Cardiovascular and infection-related mortality, hospitalizations	30% lower all-cause mortality in HDF group (HR 0.70, 95% CI 0.53–0.92)
Ok et al., (NDT 2013) [87]	Turkish Multicenter RCT	Prevalent End Stage Kidney Disease Patients	782 (391 HD, 391 HDF)	Sub 17.2 ± 1.3 Conv 19.5 ± 1.5	Comparison of postdilution online HDF vs. high-flux hemodialysis in young ESKD patients	Composite of all-cause mortality and nonfatal cardiovascular events	Cardiovascular mortality, hospitalization rates	No difference in primary outcome Better survival in high-efficiency OL-HDF subgroup (HR 0.54, 95% CI 0.31–0.93)
Morena et al., (KI 2017) [58]	French Multicenter RCT	Elderly Prevalent End Stage Kidney Disease Patients	381 (191 HD, 190 HDF)	Sub 20 ± 1.5 Conv 21 ± 1.8 L/ses	Comparison of postdilution online HDF vs. high-flux HD in elderly ESKD patients	Intradialytic tolerance	Quality of life, cardiovascular risk biomarkers	Fewer intradialytic symptoms in HDF vs HD. No significant difference in mortality or quality of life,
Blankestijn et al., (NEJM 2023) [22]	Multinational RCT	Prevalent End Stage Kidney Disease Patients	1360 (677 HD, 683 HDF)	Sub 23 ± 1 Conv. 25.5 ± 2 L/ses	Comparison of high-dose postdilution HDF vs. conventional high-flux HD	Death from any cause	Cause-specific mortality, cardiovascular events, hospitalizations	HDF reduced all-cause mortality by 23% vs. HD (17.3% vs. 21.9%)

**Table 2:** Meta-analyses published between 2004 and 2024 comparing the outcomes of convective therapies or HDF versus HD in adult patients with ESKD.

Author, Journal Year	Design	Population	n Patients	Aim and Key Features	Main Outcomes
Rabindranath et al., (AJKD 2005) [15]	Systematic review of RCTs	Adult ESKD patients on maintenance dialysis	18 trials, total 588 (HD 262, HDF 326)	Comparison of convective therapies (HF, AFB, PHF, HDF) versus HD focusing on mortality and CV outcomes	No clear mortality benefit. Differences in dialysis adequacy and solute clearances
Susantitaphong et al., (NDT 2013) [92]	Systematic review and meta-analysis of RCTs	Adult ESKD patients on maintenance dialysis	65 RCTs, total 12,182 HD 3987, HDF 2629, HF 191, AFB 505	Comparison of convective therapies (HF, AFB, PHF, HDF) versus HD focusing on mortality and CV outcomes	Reduced CV mortality, Intradialytic hypotension, and better solute clearance in HDF/HD
Nistor et al., (AJKD 2014) [93]	Systematic review and meta-analysis of RCTs	Adult ESKD patients on maintenance dialysis	35 trials, total 4039	Comparison of convective therapies (HF, AFB, PHF, HDF) vs HD focusing on mortality and CV outcomes	No mortality benefit but possible reduction in CV mortality and hypotension events in HDF/HD
Mostovaya et al., (SDI 2014) [99]	Systematic review and meta-analysis	Adult ESKD patients on maintenance dialysis	6 RCTs, total 2972 HD 1654 HDF 1318	Comparison of HDF versus HD focusing on mortality and CV outcomes related to convection volume	Reduced mortality linked to higher convection volumes in HDF/HD
Bignardi et al., (HDI 2024) [94]	Systematic review and meta-analysis of RCTs	Adult ESKD patients on maintenance dialysis	5 RCTs, total 4143 (HD 1747, HDF 1758)	Comparison of HDF versus HF-HD focusing on mortality and CV outcomes	Reduced all-cause and CV mortality in HDF/HD
Guimaraes et al., (KM 2024) [95]	Systematic review and meta-analysis of RCTs	Adult ESKD patients on maintenance dialysis	5 RCTs, total 4143 (HD 2065, HDF 2078)	Comparison of HDF versus HD focusing on mortality and CV outcomes	Reduced all-cause, CV, and infection-related mortality with HDF/HD
Vernooij et al., (Lanc 2024) [7]	Individual patient data meta-analysis of RCTs	Adult ESKD patients on maintenance dialysis	5 RCTs, total 4153 HD 2046; HDF 2050	Comparison of HDF versus HD focusing on mortality and CV outcomes and convective dose-response relationships.	Reduced all-cause mortality and CV mortality. Dose-response effect with convection volume.
Wang et al., (AJKD 2014) [96]	Systematic review and meta-analysis of RCTs	Adult ESKD patients on maintenance dialysis	16 RCTs, total 4820 HD 1520, HF 1230, AFB 980, HDF 1090	Comparison of convective therapies (HF, AFB, PHF, HDF) versus HD focusing on mortality, CV outcomes and dialysis adequacy	No clear mortality benefit. Reduced intradialytic hypotension. Improved solute clearance with HDF/HD
Silvinato et al., (2024) [97]	Systematic review and meta-analysis of RCTs	Adult ESKD patients on maintenance dialysis	6 RCTs, total 3629 HD 1808, HDF 1821	Comparison of HDF versus HD focusing on mortality and CV outcomes	Reduced all-cause and CV mortality, no effect on infection-related mortality with HDF/HD
Zhu et al., (BMCN 2024) [98]	Meta-analysis of RCTs	Adult ESKD patients on maintenance dialysis	10 RCTs, total 5120 HD 2560 HDF 2560	Comparison of HDF vs HD, with convection volume analysis	Reduced all-cause and CV mortality with high volume HDF/HD

**Table 3:** Individual patient data meta-analysis of the four main European RCTs comparing the outcomes of HDF and HD in ESKD adult patients (n = 2793 patients; HD 1369, HDF 1367): A: hazard ratios for all-cause and cause-specific mortality; B: hazard ratios for all-cause and cause-specific mortality stratified by delivered BSA-adjusted convection volume.

**(A)**					
**Cause**	**HD Events**	**HD Events/100 PY**	**HDF Events**	**HDF Events/100 PY**	**HR (95% CI)** **for HDF vs. HD**
n 2793: HD 1369 HDF 1367					
All-causes	410	12.1	359	10.45	0.86
					(0.75; 0.99)
Cardiovascular Disease	164	4.84	128	3.73	0.77
					(0.61; 0.97)
Infections	77	2.27	73	2.13	0.94
					(0.68; 1.30)
Sudden death	56	1.65	56	1.63	0.99
					(0.68; 1.43)
**(B)**					
**Cause**		**Adjusted HR (95% CI)**			
n 2793: HD 1369 HDF 1367					
BSA-Adjusted Convection Volume (L/session)		<19	19–23		>23
All-causes		0.83	0.93		0.78
		(0.66; 1.03)	(0.75; 1.16)		(0.62; 0.98)
Cardiovascular		0.92	0.71		0.69
		(0.65; 1.30)	(0.49; 1.03)		(0.47; 1.00)
Infections		1.5	0.97		0.62
		(0.92; 2.46)	(0.54; 1.74)		(0.32; 1.19)
Sudden death		1.09	1.04		0.69
		(0.69; 1.74)	(0.63; 1.70)		(0.39; 1.20)

**Table 4:** Updated individual patient data meta-analysis of RCTs comparing the outcomes of HDF (n 2050) and HD (n 2046) in ESKD adult patients.

Outcome	HD (n)	HD Events	HD Events per 100 PY	HDF (n)	HDF Events	HDF Events per 100 PY	Hazard Ratio (95% CI)
Number of patients included: n 4096: HD 2046; HDF 2050							
All-cause mortality (primary outcome)	2046	559	11.18	2050	477	9.37	0.84 (0.74–0.95)
Cardiovascular mortality	1979	202	4.04	1972	160	3.14	0.78 (0.64–0.96)
Cardiac causes	1979	117	2.34	1972	80	1.57	0.67 (0.50–0.89)
Non-cardiac causes	1979	32	0.64	1972	39	0.77	1.20 (0.75–1.91)
Unclassified	1979	53	1.06	1972	41	0.83	0.78 (0.52–1.17)
Infection-related mortality, including COVID-19	1677	118	2.36	1691	96	1.89	0.80 (0.61–1.04)
Infection-related mortality, excluding COVID-19	1677	97	1.94	1691	81	1.59	0.82 (0.61–1.10)
Sudden death	1979	98	1.96	1972	84	1.65	0.84 (0.63–1.12)
Transplantation	2070	162	2.8	2083	193	2.98	1.14 (0.92–1.41)
